# Supplementary material for: A Combined Risk Score Model to Assess Prognostic Value in Patients with Soft Tissue Sarcomas
Source: Cells. 2022 Dec 16;11(24):4077. doi: 10.3390/cells11244077 (PMC9776565; doi:10.3390/cells11244077)
Supplement: Supplementary file 1 [file cells-11-04077-s001.zip › Supplementary Tables S1-S4.pdf]

Supplementary Table S1

The WGCNA was performed on TCGA-SARC to study the hub genes.

Gene	Coef
LIPT1	-0.479559011
GCSH	0.368286205
ATP7B	0.119080216
NCOA6	0.177100002
PRPF4B	0.335911194

Note: The signature genes was used for constructing LASSO regression.

Supplementary Table S2

Patient Characteristics

Characteristics	Patients	
	n	(%)
Age, year		
< 50	15	45.45
> 50	18	54.55
Sex		
Male	19	57.58
Female	14	42.42
Site		
Extremities	24	72.73
Head, neck	2	6.06
Trunk wall	4	12.12
Internal trunk*	3	9.09
Tumor size (cm)		
< 5	9	27.27
5 < S < 10	10	30.30
>= 10	14	42.42
Tumor depth		
Superficial	8	24.24
Deep	25	75.76
TNM classification		
I	8	24.24
II	5	15.15
III	16	48.48
IV	4	12.12

Retroperitoneal space,
ntraabdominal area, and pelvis

Supplementary Table S3

The primer sequences of genes.

Gene Name	Forwand (5' - 3')	Reverse (5' - 3')
FDX1	TTCAACCTGTCACCTCATCTTTG	TGCCAGATCGAGCATGTCATT
LIPT1	CCTCTGTTGTAATTGGTAGGCAT	CTGGGGTTGGACAGCATTCAG
LIAS	CAGCCCAGTCAGACCGTTAAG	TTTCTGGCGTTTTAGGTTTCCT
DLD	CTCATGGCCTACAGGGACTTT	GCATGTTCCACCAAGTGTTTCAT
DBT	CAGTTCGCCGTCTGGCAAT	CCTGTGAATACCGGAGGTTTTG
GSCH	GAACGTCCCATCAGTACTACAT	TTCTGAAGGGTTACTCAGTGTC
DLST	GAACTGCCCTCTAGGGAGAC	AACCTTCCTGCTGTTAGGGTA
DLAT	CCGCCGCTATTACAGTCTTCC	CTCTGCAATTAGGTCACCTTCAT
PDHA1	TGGTAGCATCCCGTAATTTTGC	ATTCGGCGTACAGTCTGCATC
PDHB	AAGAGGCGCTTTCACTGGAC	ACTAACCTTGTATGCCCCATCA
SLC31A1	GGGGATGAGCTATATGGACTCC	TCACCAAACCGGAAAACAGTAG
ATP7B	CGGTTTAGTGGATATTTTGTCCC	CTCTGAACAACACCAAAATCGA
ATP7B	GCCAGCATTGCAGAAGGAAAG	TGATAAGTGATGACGGCCTCT
NCOA6	TACCAACGCAAATAAACCGAAG	TTTAGGGCCTGAGTTATCCAAG
PRPF4B	CAGCATCTTTGTCTGGTATTCG	CCAGGAACAACTGCTGACTATA
GAPDH	TATCGTGATGCTAGTCCGATG	TGCAGCTAGCTGCATCGATCGG

Supplementary Table S4

The charcteristic of 255 STS patients.

**Characteristics**		High-risk n=127	Low-risk n=128	*P* value
Age (mean (SD))		58.98 (15.77)	61.70 (12.70)	0.129
Race (%)	Asian	3 (2.4)	2 (1.6)	0.702
	Black or african american	11 (8.7)	7 (5.5)	
	Not reported	5 (3.9)	4 (3.1)	
	White	108 (85.0)	115 (89.8)	
Gender (%)	Female	73 (57.5)	66 (51.6)	0.410
	Male	54 (42.5)	62 (48.4)	
Primary diagnosis (%)	Abdominal fibromatosis	1 (0.8)	0 (0.0)	0.200
	Aggressive fibromatosis	1 (0.8)	0 (0.0)	
	Dedifferentiated liposarcoma	26 (20.5)	30 (23.4)	

	Fibromyxosarcoma	12 (9.4)	13 (10.2)	
	Giant cell sarcoma	3 (2.4)	0 (0.0)	
	Leiomyosarcoma, NOS	41 (32.3)	59 (46.1)	
	Liposarcoma, well differentiated	1 (0.8)	0 (0.0)	
	Malignant fibrous histiocytoma	5 (3.9)	7 (5.5)	
	Malignant peripheral nerve sheath tumor	7 (5.5)	2 (1.6)	
	Myxoid leiomyosarcoma	2 (1.6)	1 (0.8)	
	Pleomorphic liposarcoma	1 (0.8)	0 (0.0)	
	Synovial sarcoma, biphasic	2 (1.6)	0 (0.0)	
	Synovial sarcoma, NOS	1 (0.8)	1 (0.8)	
	Synovial sarcoma, spindle cell	4 (3.1)	2 (1.6)	
	Undifferentiated sarcoma	20 (15.7)	13 (10.2)	
Metastatic diagnosis (%)	Not reported	35 (27.6)	46 (35.9)	0.129
	NO	58 (45.7)	60 (46.9)	
	YES	34 (26.8)	22 (17.2)	
Tumor total necrosis percent (%)	Not reported	34 (26.8)	43 (33.6)	0.689
	<10% (focal necrosis)	21 (16.5)	16 (12.5)	
	0% (no necrosis or no mention of necrosis)	36 (28.3)	33 (25.8)	
	Extensive Necrosis (>50%)	7 (5.5)	5 (3.9)	
	Moderate Necrosis (>=10, <50%)	29 (22.8)	31 (24.2)	
Tumor depth (%)	Not reported	28 (22.0)	24 (18.8)	0.686
	Deep	90 (70.9)	92 (71.9)	
	Superficial	9 (7.1)	12 (9.4)	

Note: $P < 0.05$ was considered to be significant difference. STS: soft tissue sarcoma
